# A Hand-Guided Robotic Drill for Vestibular Implant Surgery—Feasibility of Preventing Membranous Labyrinth Rupture

**DOI:** 10.1177/19160216261433549

**Published:** 2026-05-08

**Authors:** Joost Johannes Antonius Stultiens, Xinli Du, Jérôme Joseph Waterval, Angélica Pérez Fornos, Nils Guinand, Raymond van de Berg

**Affiliations:** 1 Department of Otorhinolaryngology-Head and Neck Surgery, School for Mental Health and Neuroscience, Faculty of Health Medicine and Life Sciences, Maastricht University Medical Center, Maastricht, The Netherlands; 2 Department of Mechanical and Aerospace Engineering, College of Engineering, Design and Physical Sciences, Brunel University of London, Uxbridge, UK; 3 Division of Otorhinolaryngology-Head and Neck Surgery, Department of Clinical Neurosciences, Geneva University Hospitals, University of Geneva, Geneva, Switzerland

**Keywords:** vestibular implant, semicircular canals, inner ear, prosthesis implantation, implanted electrodes, surgical procedures, robotics, robot-assisted surgery, feasibility studies, bilateral vestibulopathy

## Abstract

**Importance:**

Progress in vestibular implantation offers hope for patients with bilateral vestibulopathy. However, surgically opening the semicircular canals risks breaching the membranous labyrinth, which may induce sensorineural hearing loss. A robotic drill sensing force and torque might prevent membranous labyrinth rupture.

**Primary objective:**

To assess the feasibility of force- and torque-based automatic cessation in a hand-guided robotic drill for fenestrating the bony semicircular canals without rupturing the membranous labyrinth.

**Secondary objective:**

To fit an electrode dummy through the fenestrations.

**Design:**

Feasibility study using human cadaveric temporal bones.

**Setting:**

Laboratory.

**Participants:**

Ten formalin-fixed human temporal bones.

**Intervention:**

After performing a cortical mastoidectomy and skeletonizing the semicircular canals, a hand-guided robotic drill was used to drill 2 fenestrations in each semicircular canal. A silicone electrode dummy was inserted through each fenestration.

**Main outcome measures:**

Proportion of fenestrations with intact membranous labyrinth, as evaluated with a surgical microscope. Proportion of fenestrations allowing electrode insertion without additional manipulation.

**Results:**

A total of 60 fenestrations were made in 30 semicircular canals from 10 temporal bones. Technical issues related to drill bit fixation occurred in 6 fenestrations. The remaining 54 fenestrations were all made without visible damage to the membranous labyrinth. In 81% of these fenestrations (44/54), the electrode could be advanced without requiring additional manipulation. The technical issue was related to improper alignment of the drill bit, leading to incorrect force and torque sensing.

**Conclusions:**

Force- and torque-based automatic cessation in a hand-guided robotic drill is feasible for fenestrating the bony semicircular canals without rupturing the membranous labyrinth. However, improved burr fixation is required for consistent and reliable performance.

**Relevance:**

The investigated approach holds potential to improve safety and precision in semicircular canal surgery, such as vestibular implantation. This may expand treatment options for patients with residual inner ear function.

## Graphical abstract

**Graphical Abstract:**
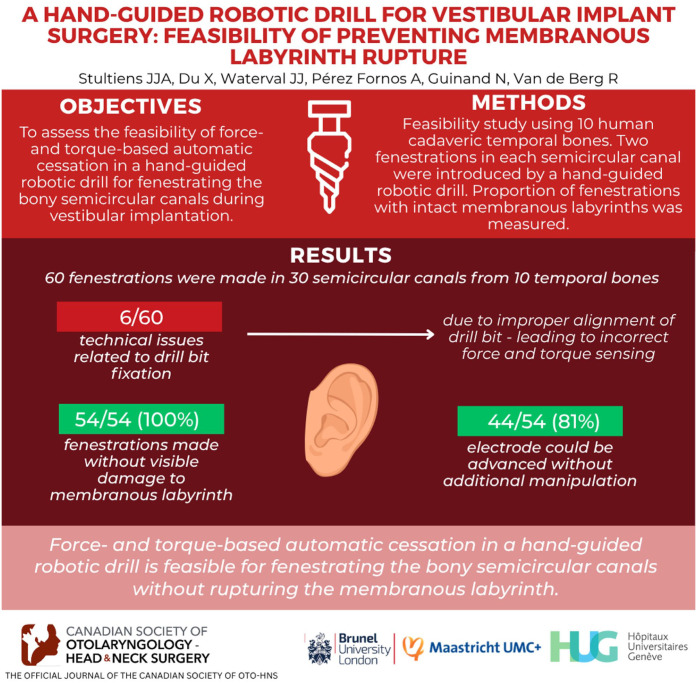

## Key Message

- A hand-guided robotic drill with force and torque sensing was set up to detect endosteal breakthrough and achieved controlled fenestration without membranous labyrinth rupture (54/54).
- A silicone electrode dummy advanced through 81% of fenestrations without additional manipulation, demonstrating practical feasibility.
- The approach can facilitate preservation of the endolymphatic compartment and, with minor refinement of drill bit attachment, holds strong potential for vestibular implantation and other inner ear surgeries in patients with residual inner ear function.

## Introduction

Advances made in the development of a vestibular implant are promising. This prosthesis is designed to aid patients with bilateral vestibulopathy, for which no cure is available yet. Due to the bilaterally reduced or absent vestibular function, these patients suffer from several disabling symptoms, such as chronic dizziness, imbalance, and oscillopsia.^1^ This disorder was estimated to affect 28 in 100,000 adults in the United States of America,^2^ with an annual economic burden of $13,019 ($0-$48,830) per patient,^3^ emphasizing the potential individual and societal benefit of a treatment.

The vestibular implant transmits motion information from gyroscopes and/or accelerometers to the vestibular nerve fibers using an electric signal.^4-6^ When delivered to the ampullary nerves of the semicircular canals, this neuroprosthesis can partially restore vestibular reflexes, such as the vestibulo-ocular reflex.^4,6,7^ It can also provide functional improvements,^8,9^ and increase quality of life.^9^ At present, implantation is mostly performed using the intralabyrinthine surgical technique. In this approach, the electrode leads are inserted in the semicircular canals through a fenestration drilled in the bony semicircular canals. The electrodes are intended to be placed in the semicircular canal ampullae, in the vicinity of the ampullary nerves.^10^ However, correct electrode placement is a challenge^11,12^ and there is a risk of sensorineural hearing loss.^6,9,12^

Because of this risk, patient selection in vestibular implantation trials is currently often limited to patients with profound sensorineural hearing loss in at least 1 ear. However, more than half of the patients with bilateral vestibulopathy do not suffer from severe hearing loss.^13^ Only selecting patients with severe to profound hearing impairment restricts the potential benefits of a vestibular implant to a minority of the patient population. It is therefore important to develop a vestibular neuroprosthesis and surgical approach for implantation with a low risk of significantly compromising hearing.

Deterioration of hearing in vestibular implantation could be related to leakage of endolymph during the surgical procedure. This may occur after the membranous labyrinth ruptures when the fenestration in the bony semicircular canal is drilled. When the semicircular canal is opened, but the membranous labyrinth is kept intact, sensorineural hearing may remain unaffected.^14,15^ Hearing, assessed by Auditory Brainstem Responses, was preserved intraoperatively during vestibular implantation in a patient where no leakage of endolymph was observed.^16^ Since leakage of endolymph could compromise residual inner ear function, limiting this risk might be beneficial for future potential vestibular implant candidates with residual hearing.

Previously, a robotic micro-drill was developed, which might minimize the risk of endolymphatic leakage during vestibular implantation. This drill was initially developed for stapedotomy^17^ and cochleostomy^18^ procedures, to minimize penetration of the drill through the inner surface of the bone. It relies on force and torque feedback to simultaneously determine the state of the process and automatically stop drilling if an undesired medium for drilling is detected.^19^ It was developed to be used as a system supported by a mechanical arm or as hand-guided. Various drill bits can be used.^20^ In cochlear implant surgery, this drill minimized disturbances of the endosteum when drilling a porcine cochleostomy, compared to manual drilling.^21^ It was also able to drill a human cochlea while leaving the endosteal membrane intact.^22^ It was hypothesized that this drill could also fenestrate the bony semicircular canals without damaging the membranous labyrinth. As a result, leakage of endolymph during vestibular implantation could be prevented.

Therefore, the aim of this study was to test the feasibility of the hand-guided version of this robotic drill to fenestrate the bony semicircular canals without rupturing the membranous labyrinth. A secondary objective was to test whether a silicone electrode dummy could fit through the created fenestrations. After all, fenestrations should allow electrode insertion into the semicircular canals.

## Materials and Methods

### Materials

This study was performed using formalin-fixed cadaveric human temporal bones. These were prepared for the study by using a regular otologic drill to perform a cortical mastoidectomy and expose the 3 bony semicircular canals. These bony canals were then skeletonized (“bluelined”) to visualize the course of the canal. Next, a small indentation was made with a 2.0 mm diamond burr at the intended fenestration sites (2 per semicircular canal), without opening the canal. This created a starting point for the robotic drilling trajectory and prevented it from slipping from the surface of the canal.

#### Robotic Drill

**Figure 1.**
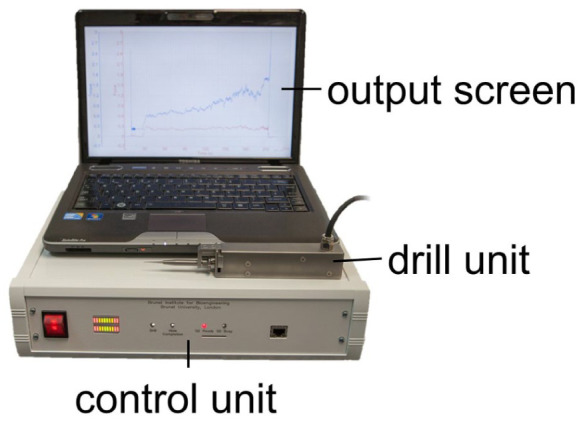
The experimental hand-guided surgical robot drill system.

**Figure 2.**
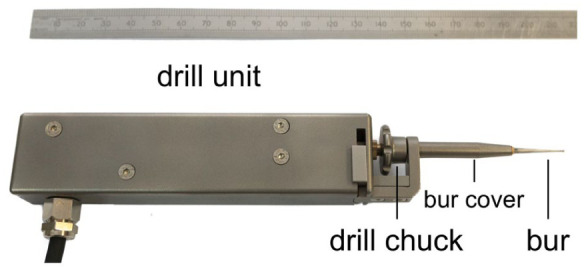
The robotic drill unit, with a bur installed.

**Figure 3.**
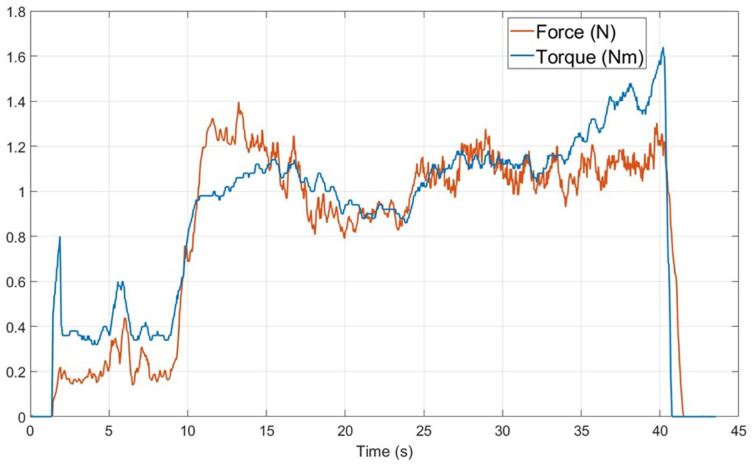
Example of force and torque transients versus time during robotic drilling of a semicircular canal, as presented on the output screen (case temporal bone 5).

The hand-guided drilling system (Figure 1) comprises 3 essential units: the drill unit (detailed photograph in Figure 2), the hardwired control unit, and an output screen. The design of the drill chuck enables quick changes of most commercially available burrs or drill bits and ensures efficient transfer of pushing force to the internal sensor. The hardwired control unit consists of 2 micro-controllers. One micro-controller is responsible for servo control of the drill unit, the other one manages information communication with the output screen via ethernet. LED bars on the control unit indicate the pushing force during drilling, with the optimal range displayed as a green area. The output screen displays a user interface, which presents information such as pushing force, rotation torque, and rotation speed. The drill system detects breakthrough from bone to the endosteum through a sudden decrease in axial force and a simultaneous increase in torque. These changes, caused by the transition from dense bone to the less resistant endosteum, are measurable via integrated force and torque sensors (illustrated in Figure 3).

### Experimental Procedures

A 2.0 mm ball-shaped fine diamond burr (Medtronic Xomed, Jacksonville, FL, USA) was installed in the robotic drill chuck. This drill bit size was determined based on a pilot trial. This trial investigated the optimal settings and equipment to drill a hole in the semicircular canal with a sufficient size (diameter of at least 0.8 mm). Drill speed was set on 2000 rotations per minute (rpm). The surgeon positioned the drill in the indentation, such that it was approximately perpendicular to the canal. Then, the drill was released from the surface, the drill was activated, and contact with the bony surface was reestablished to start drilling. When the drilling automatically stopped, the drill was removed from the surgical site. Water was splashed on the created hole, in order to flush away most of the bone dust and endosteum without damaging the underlying membranous labyrinth. A small blunt pick was used to carefully sweep away residual bone dust and endosteum in the fenestration. The fenestration and the exposed membranous labyrinth were inspected with the surgical microscope (OPMI Pentero, Carl Zeiss Surgical GmbH, Oberkochen, Germany; example of the procedure in Supplemental Video 1; Video 2 shows how the membranous labyrinth can be inspected). In case the bony fenestration was deemed very small (“pinpoint”), the drilling process was restarted at the same location. Subsequently, the membranous labyrinth was inspected with the microscope and it was assessed whether this was either intact or ruptured. Two fenestrations were drilled in each semicircular canal. The order of fenestrating the canals was not fixed. After all fenestrations in the temporal bone were made, a dummy electrode (ø 0.8 mm, MED-EL, Innsbruck, Austria) was inserted through each fenestration. This electrode was made from silicone with a metal wire inside and a metal ball contact at the tip. The dummy was gently inserted to test whether the created hole was large enough to fit this electrode. If the dummy electrode did not fit, the drilling was not restarted for a new attempt.

### Analysis

The primary outcome was the proportion of fenestrations that were made without rupturing the membranous labyrinth. A secondary outcome was the proportion of created fenestrations that were large enough to insert the dummy electrode into the canal. Descriptive statistics were used to assess these outcomes. Analyses were performed on the fenestrations in which the drill did not show any technical issues. The fenestrations made while a drill-related technical issue occurred were described separately.

### Ethics

This study was conducted thanks to anatomical donors. Handwritten and signed codicils from these donors are maintained at the Department of Anatomy and Embryology, in compliance with the Dutch Corpse Disposal Act (“Wet op de lijkbezorging,” 1991). The procedures undertaken in this investigation adhered to the legislation and ethical standards in the Netherlands.

## Results

A total of 60 fenestrations were made in 30 semicircular canals from 10 temporal bones. Five left and 5 right temporal bones were included. The fenestrations were drilled by 3 different surgeons, each drilling 5, 4, or 1 temporal bone(s). In 6 fenestrations (in 2 temporal bones), a technical issue was identified (see below).

**Figure 4.**
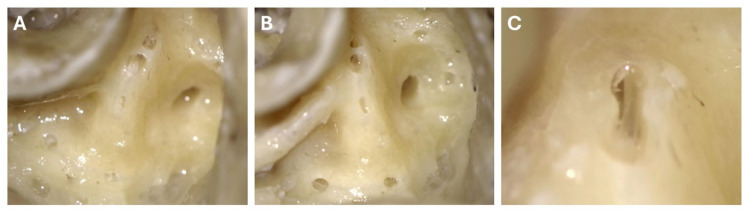
Examples of semicircular canal fenestrations, shown on temporal bone #8, after drilling with the robotic hand-guided surgical drill. (A) Fenestrations in the 3 semicircular canals. The “endosteal cap” is visible, for example on the membranous labyrinth of the lateral semicircular canal. (B) After removal of the endosteal cap, the intact membranous labyrinth is visible through multiple fenestrations. (C) Two fenestrations of the lateral semicircular canal were connected to visualize the intact membranous labyrinth in more detail.

The 54 fenestrations without technical issues were all made without visible damage to the membranous labyrinth (19 in superior and posterior canals, 16 in lateral canals; Table 1 and Figure 4). The membrane could be properly inspected, sometimes requiring the manual removal of a thin osseous-like layer (“endosteal cap”; Figure 4). An example procedure is demonstrated in Supplemental Video 1 (fenestration #1 in temporal bone 5). In half of the cases, the hole was not created or only very small when the drill stopped after the first run. In these cases, 2 runs (15 cases, 28%), 3 runs (7 cases, 13%), or 4 runs (5 cases, 9%) were necessary to create a larger fenestration.

**Table 1.** The Success of Fenestrating the Semicircular Canals Using the Hand-Guided Robotic Drill.

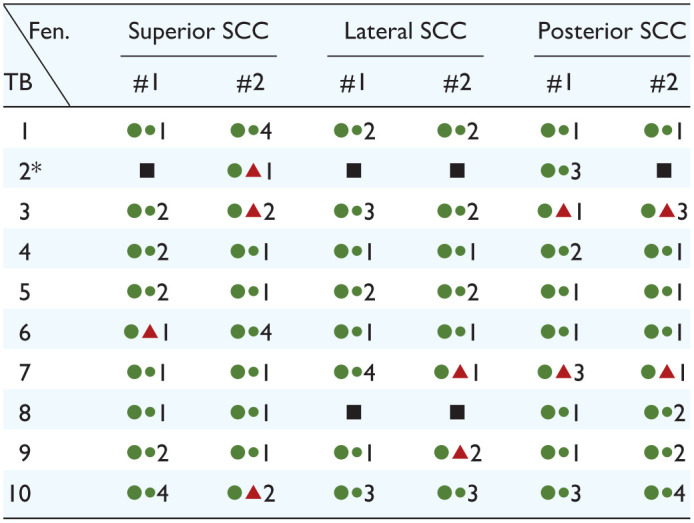

Each semicircular canal was fenestrated twice. For each fenestration, the first large symbol represents the success in preserving the membranous labyrinth, and the second smaller symbol represents the success of inserting the dummy electrode. Green circle: successful; red triangle: unsuccessful; black square: technical issue. Third is the number of times that the automatic drilling process was run before a proper fenestration was created.

Abbreviations: Fen., fenestration; SCC, semicircular canal; TB, temporal bone.

* Note that the chronological order was different for each temporal bone.

The technical issues in temporal bone 2 occurred successively as first the posterior, then the lateral, and then the superior SCC were fenestrated.

The electrode could be advanced in 44 of the 54 fenestrations (81%). Hence, in 19%, the hole was eventually not large enough to facilitate electrode insertion without extra manipulation.

A technical issue occurred while drilling 6 of the fenestrations: automatic cessation was impaired due to loosening and incorrect alignment of the drill bit, causing incorrect sensing of force and torque. This was first observed in temporal bone 2, where 1 fenestration resulted in rupture of the membranous labyrinth during enlargement after an initially successful tiny fenestration. Similar abnormal drill behavior was subsequently noted in 1 successful fenestration with an intact membranous labyrinth (and sufficient fenestration size for electrode insertion) and in 2 additional fenestrations in the same specimen, which were manually aborted while enlarging an initial tiny fenestration. Retrospectively, abnormal drilling noise was noted during these procedures, but the cause was only identified after the fourth affected fenestration. The issue recurred in temporal bone 8 in 2 fenestrations that were manually aborted due to abnormal drilling noise. The underlying cause, again improper drill bit fixation, was then identified while drilling the second affected fenestration.

## Discussion

This study investigated the feasibility of a robotic hand-guided surgical drill that relies on force and torque parameters to prevent rupture of the membranous labyrinth when opening the semicircular canals. Such a drill could be used for vestibular implantation or other surgical procedures in which endolymphatic compartment preservation is desired (eg, plugging of a semicircular canal). Of the 60 fenestrations performed, technical issues related to drill bit fixation occurred in 6 cases. In all remaining cases (54/60), the drill successfully preserved the membranous labyrinth. These results demonstrate the feasibility of this approach for limiting damage to the endolymphatic compartment when fenestrating the semicircular canals.

When considering hearing preservation in vestibular surgery, the results of this robotic drill are very promising. Force-and-torque-controlled robotic drills that drill in a linear direction^17,18^ or drill by various computed paths^23^ have been developed, especially for improving safety. As there is a high contrast in resistance between bone and the delicate structures, such drills could detect a change in drilling medium and consequently automate instant cessation when drilling towards softer structures. Specifically for the application in vestibular implant surgery, this could be very important. The vestibular part of the endolymphatic compartment is connected to the cochlear part (cochlear duct, or scala media), containing the auditory sensory organ (organ of Corti). When the endolymphatic compartment is not opened during drilling, disturbance of the cochlear duct content may be limited, thereby potentially increasing the likelihood of preserving hearing.^14,15^ Although electrode insertion might still rupture the membranous labyrinth, it could become immediately “plugged” by the electrode, limiting endolymph leakage.^24^ As the current drill may save the endosteum, perilymph leakage might also be limited until the electrode is inserted.^25^ Other factors, such as reducing pressure changes during insertion, drilling-induced acoustic energy, and inflammatory processes, may also contribute to hearing preservation.

In 44 of the 54 cases (81%), the fenestration was large enough to fit an electrode of 0.8 mm diameter. This implies that for vestibular implantation, some fenestrations should additionally be enlarged to insert electrodes. In these cases, a small hook may be used to enlarge these fenestrations, as routinely performed in semicircular canal plugging procedures.^25^

Interestingly, it was often seen that when the drill stopped, a thin layer of tissue covered the fluid-filled canal, likely a “cap” of mobilized endosteum (Figure 4). This endosteal cap could easily be removed with a blunt pick, pointed needle, or microhook, after which an intact membranous labyrinth became visible (also see Supplemental Video 1, starting from 48 seconds). Therefore, it is important to note that such a cap does not indicate membranous labyrinth damage, but might even protect the membranous labyrinth during drilling. For certain purposes, it may be necessary to remove this cap. While manual removal introduces a small risk of human error, it can generally be performed carefully without rupturing the membranous labyrinth, as its (approximate) location can be seen or inferred from the fenestration. The greater challenge lies in removing the bone while preserving the underlying membranous labyrinth, a step facilitated by the drill. Sometimes, the thin covering fragments seemed loosely attached to the membranous labyrinth, suggesting that some connective tissue strands between the membranous and bony labyrinth were still intact.

The field of robotic surgery is emerging and robotic drills have been developed for otologic applications, with various levels of autonomy.^26-29^ However, their current accuracy seems insufficient for fenestrating the semicircular canals with preservation of the membranous labyrinth. After all, the very thin bluelined semicircular canal wall and the short distance to the underlying membranous labyrinth require submillimetric accuracy. Improved registration methods, such as with bone-anchored fiducials, may enhance the accuracy.^30^ Yet, a hand-guided robotic drill facilitates the combination of the surgeon’s location precision with the sensing precision of the robotics. It gives freehand flexibility in precisely determining the drilling location and direction. Although the currently investigated device drills linearly forward, it allows for small directional changes during drilling, and the starting direction can also be determined flexibly. In a previous pilot trial, it seemed that for the current application of fenestrating a semicircular canal, the hand-guided drill was also more consistent than a fixed arm-supported version of this drill.

### Surgical Considerations

Based on experiments piloting the drill prior to this study, a few things were identified worth considering when preparing the semicircular canals for drilling with the hand-guided robotic drill and during drilling. First, the canal should be meticulously bluelined (visibility according to blueline in Supplemental Video 1), to provide a clear view of the course of the canal. As a result, the surgeon can create the fenestration more precisely in the center of the lumen of the canal. Second, the canal should be skeletonized around its circumference to create a convex shape of the bone at the blueline. This appeared to improve the drill’s ability to detect breakthroughs in the canal. Most likely the convex shape ensures that when the endosteum is reached, there is no contact between the side of the burr head and the bone. After all, due to the linear drilling trajectory, the drill can more reliably detect the lumen of the canal when it is encountered by the center of the burr. When the middle of the burr head drills in the bony side border of the canal, the measured (linear) force may still be high, but part of the burr head may already be drilling inside of the canal. Consequently, the drill would not stop at the correct moment and may rupture the membranous labyrinth. Third, it is important for the automatic detection that the (preferably perpendicular) angle between the drill and the bony canal is kept relatively constant during drilling. To facilitate this, and to prevent slippage of the burr, a small indentation can be created before starting the robotic drilling process, as described in the methods section. Fourth, after drilling for a certain duration, the surgeon may have the urge to decrease the pressure on the drill to be more safe for the membranous labyrinth. However, keeping a constant pressure on the drill is important: the drill will more reliably sense the sudden changes in force and torque, needed to automatically stop drilling. Furthermore, for the goal of creating a fenestration in the semicircular canal, previous pilot trials suggested that a ball-shaped burr of 2.0 mm diameter was best for creating a properly sized fenestration. Due to the mechanism of action, the created fenestration is always smaller than the diameter of the drill bit. This also prevents the burr head from drilling completely inside the canal. A non-spherical burr design might be beneficial to approach the tubular canal, but this should be tested in a future trial.

### Technical Failures and Device Design

Two separate times, a technical issue occurred. The first time it was only discovered after creating 4 fenestrations (in temporal bone 2). It was noticed that the drill made a louder and varying noise. Inspection of the drill unit to identify the cause of these consecutive malfunctioning attempts revealed that the burr was rotating unevenly. Reattaching the burr resolved the noise and disturbed movement. Subsequent fenestrations were completed without issue until the same problem recurred 6 temporal bones (34 fenestrations) later. It was only recognized as the same issue while drilling the second affected fenestration. Retrospectively, the noise could also be seen in the torque and force signals. Recognition of this issue at the time it occurs would improve procedural safety. The current keyless drill chuck facilitates the universal use of multiple (commercially available) types of burrs or drill bits. These are tightened by turning the chuck around the burr, but without interlocking components. The connection might have become loose while drilling the many fenestrations. Although this hampered consistent performance, redesign could address this issue. This will likely decrease burr misalignment and further decrease the likelihood of unrecognized abnormal behavior. Nevertheless, the principle could be demonstrated in the current study.

In addition, it should be noted that in about half of the fenestrations, multiple robotic activations were necessary. While ideally the drill would complete the fenestration in a single attempt and minimize surgical time, the handheld concept enabled swift reinitiation of subsequent attempts. Quantification of drilling times was outside the scope of this study, but individual activation runs were generally short (approximately 1 minute, with subsequent runs typically shorter).

### Limitations

Certain limitations were identified in this study. First of all, this study was performed on cadaveric formalin-fixed temporal bones. This may impact the susceptibility of the membranous labyrinth for rupture in 2 ways. First, the pressure in the endolymphatic compartment is likely decreased in a deceased human. Second, the fixation medium may impact the strength of the membrane. However, since the drill merely breaks the endosteum, the drill is often not in contact with the membranous labyrinth itself. Another limitation involved the method of determining damage of the membranous labyrinth. The intactness was determined by inspection with the surgical microscope. Very small tears that cannot be visualized with the surgical microscope could not be excluded. Nevertheless, by rinsing water on the fenestration and sweeping away bone dust with a blunt pick, the membranous labyrinth could be thoroughly inspected and the clear, smooth, and shiny tube could be visualized and followed without interruptions (Figure 4). Finally, the procedures were performed by 3 different surgeons, which may have introduced some inter-operator variability in applied force and handling of the device. All surgeons followed the surgical considerations described above. Nevertheless, the findings suggest that performance is not highly dependent on individual surgical style.

## Conclusions

Taken these results into account, the hand-guided robotic drill seems to be a promising tool for preservation of the membranous labyrinth when fenestrating the semicircular canals. The combined features of this robotic surgical drill could be incorporated into fully robotic otologic drills. Yet, the present study showed that these features can be used in a hand-guided drill. With adequate drill bit fixation, consistency and reliability can likely be improved. As a result, otologic drilling could become safer for delicate procedures, such as vestibular implantation.

This study demonstrates the feasibility of a robotic hand-guided surgical drill sensing force- and torque to fenestrate the semicircular canals while preserving the membranous labyrinth.
